# The virtual institution: cross-sectional length of stay in general adult and forensic psychiatry beds

**DOI:** 10.1186/s13033-015-0017-7

**Published:** 2015-06-30

**Authors:** Anand Sharma, Warren Dunn, Clare O’Toole, Harry G Kennedy

**Affiliations:** North London Forensic Mental Health Service, Chase Farm Hospital, Enfield, London, UK; Edenfield Centre, Manchester, UK; John Howard Centre, East London Foundation Trust, Hackney, E9 5TD UK; National Forensic Mental Health Service, Central Mental Hospital, Dundrum, Dublin 14, Ireland; Department of Psychiatry, Trinity College, Dublin, Ireland

**Keywords:** Length of stay, Psychiatry, Forensic, Long term, Mental health, Hospital

## Abstract

**Background:**

Length of stay in psychiatric hospitals interests health service planners, economists and clinicians. At a systems level it is preferable to study general adult and forensic psychiatric beds together since these are likely to be inter-dependent. We examined whether patients were placed according to specialist need or according to their cross-sectional length of stay.

**Methods:**

A one night census of all registered mental nursing home (RMNH) beds was carried out for a defined catchment area of 1.2 m population in north London in November 1999. This included all public sector psychiatric hospital beds, independent sector and forensic beds in and outside the catchment area. Cross-sectional length of stay was defined as time since the date of admission from the community. Log rank (Mantel-Cox) Chi squared was used to test for differences between groups and hierarchical logistic regression for statistical modelling.

**Results:**

There were 1,085 occupied psychiatric beds. Cross-sectional LOS was greater than 365 days in 43.5%. Forensic beds had longer cross-sectional LOS than general beds. LOS increased with the level of therapeutic security from open through low, medium and high secure. Cross-sectional LOS was shorter for open hospital beds than community RMNH beds, shorter for informal patients than those detained under civil mental health law, and longest for forensic detentions. Longest cross-sectional LOS were for patients placed in RMNHs in the community, 10.7% of whom were ‘forensic’ as were 25.4% of low secure patients. Designated length of stay (acute, rehab/medium term and long term) was also associated with increasing cross-sectional LOS. In regression analysis only three variables contributed to a model of cross-sectional LOS, commissioning status (general or forensic), designated length of stay and designated level of therapeutic security.

**Conclusions:**

Studying cross-sectional LOS for whole systems (all psychiatric beds) is essential for operational health service management. At the time of this survey ‘forensic’ status was the main way of accessing long term high dependency places. This has been an organic development over time, a response to patient needs rather than the outcome of any specific policy or plan.

## Background

In order to manage the use of psychiatric beds, monitoring and comparing the pattern and duration of admissions has become important. Length of stay can be measured in different ways. Length of stay is a parameter of interest to health service planners, economists and clinicians alike. Length of stay is closely related to health service costs.

For practical purposes, cross-sectional length of stay is the parameter most relevant to the day to day operational management of psychiatric services. It is used to evaluate service utilisation, as a performance indicator [1], to compare health services [2, 3] and as an outcome measure. Cross-sectional length of stay is also likely to be influenced by policy and practice.

In historical context, there has been a shift from asylum care to community care [2–5], but in some centres this has been associated with a strain on acute services [6] because of the needs of new long stay patients [7, 8]. Complex pathways have developed linking community and local mental health services with the courts [9], prisons [10] and forensic secure hospitals [11]. Because of this interdependence, when evaluating the service responses to short term, medium term and longer term patients it is necessary to consider services for both general adult and forensic psychiatric patients as a whole system.

### Therapeutic security and forensic mental health orders

During the decade prior to this study an increase occurred in the use of psychiatric beds outside the urban catchment areas. Most of this growth occurred in the use of secure psychiatric beds, both in the urban catchment area services and in ‘out of area’ services. These secure beds were usually accessed via the courts, where forensic orders could be made by judges committing a defendant to be detained in a psychiatric hospital, provided that two psychiatrists gave evidence that the patient had a mental disorder as defined in the Mental Health Act and provided that one of the psychiatrists, or an officer of the NHS could confirm that a bed was available [12]. These beds were generally designated ‘forensic’ and were provided at high secure level in three large maximum security hospitals, in medium security or low security in special regional or supra-regional units, in acute low secure units provided in district services or in open psychiatric units at district level. In practice, the environmental, relational and procedural distinctions between high, medium and low security hospitals were poorly defined at the time of this study [13] and the distinguishing characteristics of the patients requiring these levels of therapeutic security were typically described in terms of legal charges and legal category [14] with clinical characteristics and patient needs only receiving attention more recently [15].

### Rationale

We set out to study the extent to which patients in psychiatric beds were placed according to specialist need—for medium or high secure forensic care, for other specialist services such as acute intensive care or rehabilitation, or simply according to their length of stay.

### Objectives

We surveyed all psychiatric beds for a defined population of 1.2 million. We recorded designated uses for psychiatric beds and the cross-sectional length of stay on the census date. We hypothesised that the numbers of short term, medium term and longer term beds should correspond to the designated uses of actual beds for psychiatric patients following the move from the asylum to a community mental health service. We also hypothesised that cross-sectional length of stay would predominate over designated uses such as rehabilitation or forensic secure beds. An alternative model for the provision of psychiatric beds according to the need for therapeutic security (high security, medium security or low security) would either conflict with this pattern or would match it.

## Methods

### Study design

This study was approved by the local research ethics committee as a service evaluation project [16]. This study was a cross sectional survey of psychiatric bed use based on a one night census of cross-sectional length of stay for all patients in psychiatric beds for a defined catchment area in North London. A psychiatric bed was defined as a registered mental nursing home bed within the meaning of the Mental Health Act (1983) for England & Wales. The catchment area was the population of three health authorities in North London.

The Mental Health Act (1983) for England and Wales did not permit the registering of beds, wings or areas in prisons so that there were no ‘prison hospitals’ where prisoners could be treated under the Mental Health Act. The beds available in the catchment area, and the locations of patients from the catchment area who were placed outside of the catchment area, were obtained from the National Health Service commissioners of the service in order to evaluate the service by describing the use of the beds.

An individual patient’s cross-sectional length of stay on the census date was defined as the period since admission from the community, regardless of intervening placements.

### Setting

The census took place on 24th November 1999. In the month prior to the census, all locations within the catchment area identified as registered mental nursing homes were visited by AS and WD to confirm the number of places and other characteristics of the setting, and a preliminary census was completed which allowed the final census for the entire sample to be completed by visits on the day of the census itself.

Out of catchment area locations were identified from Health Authority financial returns.

A total of 40 hospital or other registered mental nursing home locations were identified within the catchment area and further afield where mentally disordered patients could be treated under the Mental Health Act or as informal (voluntary) patients. Within these, fourteen designated uses were identified, and these could be rationalised into broader categories of designated use (Tables 1, 2, 3). In addition, units were identified as commissioned for forensic or non-forensic use (Tables 4, 5) and separately as secure or open.

**Table 1:** Designated bed uses and designated length of stay

Designated bed use	N	Designated length of stay		
		Acute	Rehabilitation/medium stay	Longer stay
		N (%)	N (%)	N (%)
Acute	570	570 (100)	0	0
Rehabilitation	137	0 (0)	107 (78%)	30 (22%)
Women’s	36	33 (92)	0	3 (8)
PICU	35	35 (100)	0	0
RMNH community	43	0	0	43 (100)
Long stay hospital	73	0	0	73 (100)
Eating disorder	13	13 (100)	0	0
Challenging behaviour	12	0	12 (100)	0
Drugs and alcohol/mother and baby	9	9 (100)	0	0
NHS medium secure	54	0	54 (100)	0
Independent sector medium secure	32	0	0	32 (100)
High secure	71	0	0	71 (100)
Total	1,085	660 (61)	173 (16)	252 (23)

*PICU* psychiatric intensive care unit, *RMNH* registered mental nursing home (community unit), *NHS* National Health Service (public sector).

**Table 2:** Designated bed uses and designated level of therapeutic security

Designated bed use	N	Designated level of therapeutic security			
		Open	Low secure	Medium secure	High secure
Acute	570	570 (100)	0	0	0
Rehabilitation	137	137 (100)	0	0	0
Women’s	36	33 (92)	3 (8)	0	0
PICU	35	0	35 (100)	0	0
RMNH community	43	43 (100)	0	0	0
Long stay hospital	73	60 (82)	13 (18)	0	0
Eating disorder	13	13 (100)	0	0	0
Challenging behaviour	12	0	12 (100)	0	0
Drugs and alcohol/mother and baby	9	9 (100)	0	0	0
NHS medium secure	54	0	0	54 (100)	0
Independent sector medium secure	32	0	0	32 (100)	0
High secure	71	0	0		71 (100)
Total	1,085	865 (80)	63 (6)	86 (8)	71 (6)

*PICU* psychiatric intensive care unit, *RMNH* registered mental nursing home (community unit), *NHS* National Health Service (public sector).

**Table 3:** Designated bed uses by designated length of stay and designated level of therapeutic security

Designated uses		Designated levels of therapeutic security			
		Open	Low	Medium	High
Acute	660	625 (95)	35 (5)	0	0
Rehabilitation	173	107 (62)	12 (7)	54 (31)	0
Longer stay	252	133 (53)	16 (6)	32 (13)	71 (28)
Total	1,085	865 (80)	63 (58)	86 (79)	71 (7)

Pearson X^2^ = 461.5, df = 6, p < 0.001.

**Table 4:** Designated bed uses and designated general or forensic management, and legal category

	n	Management commissioning		Legal category Mental Health Act			
		General	Forensic	Informal	Civil MHA	Forensic unrestricted	Forensic restricted
Acute	570	570 (100)	0	346 (60)	214 (38)	9 (2)	0
Rehabilitation	137	137 (100)	0	110 (80)	26 (19)	0	1 (1)
Women’s	36	33 (92)	3 (8)	24 (66)	10 (28)	1 (3)	1 (3)
PICU	35	35 (100)	0	12 (34)	19 (54)	3 (9)	1 (3)
RMNH community	43	30 (70)	13 (30)	29 (67)	1 (2)	4 (9)	9 (21)
Long stay hospital	73	60 (82)	13 (18)	48 (66)	19 (26)	2 (3)	4 (5)
Eating disorder	13	13 (100)	0	12 (92)	1 (8)	0	0
Challenging behaviour	12	12 (100)	0	12 (100)	0	0	0
Drugs and alcohol/mother and baby	9	9 (100)	0	9 (100)	0	0	0
NHS medium secure	54	1 (2)	53 (98)	0	9 (17)	5 (9)	40 (74)
Independent medium secure	32	0	32 (100)	0	18 (56)	3 (9)	11 (34)
High secure	71	0	71 (100)	0	7 (10)	7 (10)	57 (80)
Total	1,085	900 (83)	185 (17)	602 (55)	324 (30)	34 (3)	124 (11)

Designated bed uses, commissioning (general or forensic) and legal status.

*PICU* psychiatric intensive care unit, *RMNH* registered mental nursing home (community unit), *NHS* National Health Service (public sector).

**Table 5:** Designated bed uses by designated length of stay and Mental Health Act status

Designated uses		Mental Health Act status/legal category			
		Informal N (%)	Civil N (%)	Forensic unrestricted N (%)	Forensic restricted N (%)
Acute	659	403 (61)	243 (36)	12 (2)	1 (0.0)
Medium stay/rehabilitation	173	94 (54)	33 (19)	5 (3)	41 (24)
Longer stay	252	105 (42)	48 (19)	17 (7)	82 (33)
Total	1,084	602 (56)	324 (29)	34 (3)	124 (11)

Note that ‘rehabilitation’ is here taken to mean a medium length of stay. Pearson X^2^ = 248.3, df = 6, p < 0.001.

### Participants

All those resident on the census date in a hospital or special hospital as defined in Section 145 of the Mental Health Act 1983 or in a mental nursing home registered in pursuance of the Registered Homes Act 1984 Section 22 [17] were counted. These were places where a mentally disordered person could be detained under the Mental Health Act 1983, whether under Part II the civil provisions of the Act or Part III the criminal proceedings of the Act. At the material time, for Part III patients, referred to here as forensic patients, the courts and Home Secretary (minister of justice) could specify the hospital to which a patient was committed in order to set a level of security. The Home Secretary also controlled permission for leave from the hospital, whether accompanied or unaccompanied. The treating consultant psychiatrist could not discharge a patient subject to a restriction order imposed by a criminal court, conditional or absolute discharge could only be granted by a Mental Health Review Tribunal.

### Variables

For each resident identified, in addition to the data describing the location, data were collected on the person’s recorded date of birth, gender, ethnicity (self-defined), date of admission and legal status on the date of the census and leave status. Diagnosis was not recorded and all data were anonymised from the point of collection onwards.

### Data sources

Data were obtained from the commissioners of psychiatric services on registered mental nursing homes and all such places where catchment area patients were placed outside of the catchment area. In each identified location, the anonymised individual data were obtained from paper records locally.

### Bias

No beds were omitted, though there were a number of empty beds on the census night. Only ‘occupied’ beds were included in calculations of cross-sectional length of stay. Those on leave but not discharged on the census night were however included in the calculation of cross-sectional length of stay.

### Study size

This study was a survey of all adult psychiatric beds for a geographically defined population of 1.2 million.

### Quantitative variables

Cross-sectional length of stay was calculated as the time in days since admission from the community. Changes in location during that time and changes in legal status were not the basis for calculation of cross-sectional length of stay. Cross-sectional length of stay was aggregated as the median or as mean with standard deviation and 95% confidence intervals. The 20th centile, 50th centile (median) and 80th centiles were taken as indicators of cross-sectional length of stay with the 80% centile as an arbitrary, normative indicator of ‘long stay’ status. However the percentage of patients who had been in-patients for over 12 months and the percentage who had been in-patients for over 5 years were also taken as indicators of long-stay based on clinical rather than normative grounds.

Legal status was recorded as informal or according to the section of the Mental Health Act that applied on the census date. Legal status was conflated for analysis into informal, civil or forensic and forensic was sub-divided into unrestricted and restricted.

Location was recorded first according to the ward or area of a hospital or registered mental nursing home, then conflated for analysis into designated use (Tables 1, 2, 3, 4, 5).

### Statistical methods

All data were entered in IBM SPSS v21 [18]. Cross-sectional length of stay was treated as a dependent variable. Because cross-sectional length of stay was not normally distributed the log rank (Mantel-Cox) Chi squared (X^2^) statistic was taken as a test of significant differences between groups. The Kaplan-Meyer menu in SPSS was used to generate means, medians, 95% confidence intervals and log rank tests with all cases included. For statistical modelling, the Cox regression model was used with all cases included. Cross-sectional length of stay was treated as the dependent variable and other variables were tested for significant associations expressed as the exponent of the B coefficient (here interpreted as the odds ratio).

When comparing percentages, confidence intervals were calculated using the Confidence Interval Analysis (CIA) package [19].

## Results

### Participants

We found 1,168 registered mental nursing home places for the catchment area on the census date, including individuals placed out of the catchment area. There were 67 empty beds in the catchment area on the census night. A date of admission could not be determined for a further 16. A cross-sectional length of stay could therefore be calculated for 1,085 residents on the night of the census, or 98.5% of residents identified.

### Descriptive data

For all 1,085 patients the mean cross sectional length of stay was 966.2 days (SD 2,483.5), median cross sectional length of stay was 119 days and the mode was 5 days. The range was 0–24,633 days (67.4 years). Skewness was 5.263 (SE 0.074) indicating that the data is asymmetrically distributed with a long tail of high values and kurtosis was 34.755 (SE 0.148), together indicating that the distribution of cross sectional length of stay is not normally distributed. It is notable that 43.5% had cross sectional lengths of stay greater than 365 days.

There were 420 (39%) female and 661 (61%) male patients, with data on gender missing for four. Women were slightly older than men (women mean age 41.9 years SD 13.5, men 39.7 SD 12.9, mean difference 2.2 (95% CI 0.5 to 3.9, t = −2.6, p = 0.009). Table 6 shows that women had shorter median cross-sectional lengths of stay than men (females 79.0 days, 95% CI 56.0–101.9, males 162.0, 95% CI 121.4–202.6, log rank X^2^ = 29.7, df = 1, p < 0.001).

**Table 6:** Lengths of stay (LOS)—all are cross-sectional lengths of stay (days)

	LOS (days)	95% CI		LOS (days)	95% CI		LOS (days)	95% CI		LOS (days)	95% CI	
		Lower	Upper		Lower	Upper		Lower	Upper		Lower	Upper
Gender	Male			Female								
Number	661			420								
Mean LOS	1,196.5	987.5	1,405.5	612.3	423.4	801.2						
Median LOS	162	121.4	202.6	79.0	56.0	101.9						
General or forensic	General			Forensic								
Number	900			185								
Mean LOS	697.6	541.7	853.5	2,272.6	1,906.6	2,638.6						
Median LOS	79.0	67.2	90.8	1,367.0	965.0	1,768.9						
Open or secure	Open			Secure								
Number	869			216								
Mean LOS	725.9	563.3	888.7	1,932.4	1,612.7	2,252.2						
Median LOS	83.0	68.9	97.1	1,015.0	654.9	1,375.1						
Designated length of stay	Acute			Medium term/rehab			Long term					
Number	660			172			253					
Mean LOS (days)	105.6	91.6	120.4	974.0	705.3	1,242.7	3,204.7	2,692.5	3,716.9			
Median LOS (days)	42.0	36.4	47.6	387.0	290.6	483.4	1,692.0	1,463.4	1,920.6			
Mental health act status	Informal			civil			Forensic unrestricted			Forensic restricted		
Number	602			324			34			124		
Mean LOS	896.7	671.5	1,121.9	479.2	351.3	607.0	1,358.2	581.5	2,134.9	2,475.8	1,986.8	2,564.7
Median LOS	84.0	64.8	103.2	91.0	72.4	109.6	372.0	0	923.4	1,541.0	1,196.5	1,885.5
Level of therapeutic security	Open			Low secure			Medium secure			High secure		
Number	865			63			86			71		
Mean LOS	716.2	554.1	878.2	831.7	476.8	1,186.7	1,104.5	830.5	1,378.6	3,963.5	3,277.0	4,650.1
Median LOS	82.0	67.9	96.1	540.0	133.3	946.7	567.0	363.4	770.6	3,327.0	1,939.9	4,714.1

### Designated uses

Table 1 shows that twelve separate designated uses could be identified for psychiatric beds. These could be classified according to designated length of stay into acute, rehabilitation/medium stay and longer stay categories. Table 2 shows that the beds could also be classified according to the designated level of therapeutic security—open, low secure, medium secure and high secure. Table 3 shows that these categories according to designated length of stay and designated level of therapeutic security were associated (X^2^ = 461.5, df = 6, p < 0.001) with designated lengths of stay and designated levels of therapeutic security increasing together, though there was much overlap. Table 4 shows that beds could also be classified according to general and forensic commissioning status and for Mental Health Act category (informal, civilly detained, forensic unrestricted and forensic restricted). Table 5 shows that Mental Health Act status and designated length of stay were also associated (X^2^ = 248.3, df = 6, p < 0.001), with forensic detentions increasingly accounting for designated medium and longer stay beds.

When beds were divided into forensic or general according to commissioning, there were 900 (83%) general and 185 (17%) forensic beds, with longer mean cross-sectional lengths of stay for forensic beds (log rank X^2^ = 127.1, df = 1, p < 0.001).

### Open and secure beds

Table 6 shows there were 869 (80%) open beds and 216 (20%) secure (locked) beds, with secure beds again having a longer median cross-sectional length of stay (open beds 83.0 days, 95% CI 68.9–97.1, secure beds 1,015.0 days, 95% CI 654.9–1,375.1, log rank X^2^ = 100.2, df = 1, p < 0.001).

Further examining the levels of therapeutic security, there were 865 (80%) open beds, 63 (6%) low secure beds, 86 (8%) medium secure beds and 71 (7%) high secure beds, with progressively longer cross-sectional lengths of stay as the level of therapeutic security increased (log rank X^2^ = 123.7, df = 3, p < 0.001). Open beds could be further sub-divided into open hospital beds [n = 744 (69%), mean cross-sectional LOS 296.9 days, 95% CI 222.2–371.7, median cross-sectional LOS 55 days, 95% CI 45.5–64.5] and registered mental nursing home beds [n = 121 (11%), mean cross-sectional LOS 3,293.9 days, 95% CI 2,349.4–4,238.5, median cross-sectional LOS 1,257 days, 95% CI 971.8–1,542.2 log rank X^2^ = 187.9, df = 1, p < 0.001]. This reflects the role of the registered mental nursing home community beds in providing for the long term needs of some patients.

Further analysis showed that although open and low secure beds were significantly different in cross-sectional length of stay (log rank X^2^ = 7.1, df = 1, p = 0.008), and medium and high secure were also significantly different in cross-sectional length of stay (log rank X^2^ = 63.8, df = 1, p < 0.001), medium and low secure cross-sectional lengths of stay overlapped and were not significantly different (log rank X^2^ = 2.9, df = 1, p = 0.09).

Tables 7 and 8 show that the 80th centile for cross-sectional length of stay in an open bed was 524 days, 3,334 days in a secure bed. In open beds, 24.2% has cross-sectional LOS greater than a year, 8% greater than 5 years. For secure beds 70.8% had stays greater than a year, 33.1% greater than 5 years. Further subdivision of level of therapeutic security shows increasing proportions of long stay patients with increasing levels of therapeutic security, as well as increasing mean and median lengths of stay.

**Table 7:** Cross-sectional length of stay by designated use and legal status

	n	20th centile (days)	50th centile/median (days)	80th centile (days)	% over 1 year			% over 5 years		
					%	95% confidence interval		%	95% confidence interval	
						Lower	Upper		Lower	Upper
All	1,085	21.0	119.0	1,031.0	33.5	30.7	36.3	13	11.1	15.1
Commissioning										
General	900	17.0	80.0	497.0	23.4	20.8	26.3	17.9	15.5	20.5
Forensic	185	400.8	1,367.0	4,044.8	81.2	74.8	86.1	39.1	32.5	46.4
Open or secure										
Open	869	17.0	83.0	524	24.2	21.4	27.1	8	6.3	9.9
Secure	216	134.4	1,023.0	3,334.0	70.8	64.4	76.5	33.1	27.1	39.6
Designated length of stay										
Acute	660	12.0	42.5	142.5	6.3	4.7	8.5	0	0	0.01
Medium/rehabilitation	173	134.8	389.0	1,333.6	52.6	42.5	59.9	13.0	9.0	19.2
Long stay	252	694.6	1689.5	4,394.0	91.1	87.1	94.2	47.0	40.8	53.0
Mental Health Act status										
Informal	602	15.0	84.0	737.2	28.1	24.6	31.8	10.4	8.3	13.2
Civil detention	324	20.0	91.0	447.0	22.0	17.8	26.7	5.8	3.8	9.0
Forensic unrestricted	34	83.0	400.0	1,735.0	65.9	50.8	80.9	16.1	6.4	30.1
Forensic restricted	124	404.0	1,565.5	4,172.0	82.7	74.6	88.0	43.1	35.1	52.3
Level of therapeutic security										
Open	865	16.2	82.0	506.6	23.7	21.0	26.6	8	6.4	10.0
Low	63	50.4	540.0	1,362.6	51.6	38.8	62.7	10.3	5.5	32.3
Medium	86	167.4	571.5	1,744.6	65.1	54.6	74.3	14.4	8.2	22.8
High	71	1,540.2	3,327.0	1,930.8	96.8	90.3	99.2	91.1	82.8	96.1

**Table 8:** Cox regression for length of stay, forward stepwise, likelihood ratio

	Wald X^2^	df	p	Odds ratio	95% CI	
					Lower	Upper
Commissioning status						
General versus forensic	12.4	1	<0.001	0.483	0.322	0.726
Designated length of stay	532.2	2	<0.001			
Long stay versus acute	489.3	1	<0.001	14.3	11.3	18.1
Long stay versus medium term	58.6	1	<0.001	2.5	1.9	3.1
Designated level of security	28.9	3	<0.001			
High versus open	18.1	1	<0.001	2.6	1.7	4.1
High versus low	5.9	1	0.015	1.8	1.1	2.7
High versus medium	9.2	1	0.002	1.7	1.2	2.5

Variables gender, secure or open, designated length of stay (acute, medium term or long stay), designated level of security (open, low, medium or high security) and legal status (informal, civil, forensic).

### Legal status

The legal status of all patients was informal 602 (55%), detained under civil mental health legislation 324 (30%), detained under forensic sections of mental health legislation 158 (15%) and there was a significant difference in cross-sectional length of stay (Table 6), arising from the forensic cohort (log rank X^2^ = 92.7, df = 2, p < 0.001). Of the 158 forensic orders, 34 (3% of total) were detained without restrictions on discharge (median cross-sectional length of stay 372.0 days, 95% CI 0.0–923.4) and 124 (11% of total) were detained with restrictions on discharge (median cross-sectional length of stay 1,541.0 days, 95% CI 1,196.5–1,885.5) also a significant difference (log rank X^2^ = 7.6, df = 1, p = 0.006).

Table 7 shows that legal status was strongly associated with length of stay, with median and 80th centile lengths of stay increasing markedly in steps from informal through civil detention to forensic unrestricted and forensic restricted groups. While 28.1% of informal patients had been in hospital for a year or more and 10.4% had been in hospital for 5 years or more, this increased progressively with 74.6% of forensic restricted patients in hospital for a year or more and 43.1% in hospital for 5 years or more.

### Outcome data

#### Acute, medium and long term

When the beds are divided according to their designated use as acute (n = 660, 61%), rehabilitation/medium term (n = 172, 16%) or long-stay (n = 253, 23%), there was a significant difference between cross-sectional median lengths of stay (log rank X^2^ = 801.6, df = 2, p < 0.001) as expected. Table 7 confirms this pattern with increasing proportions of long stay patients and increasing cross-sectional lengths of stay at the median and 80th centiles.

#### Open or secure

Further exploring the relationship between levels of therapeutic security and cross-sectional length of stay, there were 865 (80%) beds at open or minimal security, low security 63 beds (6%), medium security 86 beds (8%) and high security 71 beds (7%). Cross-sectional median lengths of stay differed significantly (log rank X^2^ 123.7, df = 3, p < 0.001). As before, Table 7 confirms this trend with increasing cross-sectional lengths of stay at the median and 80th centiles as the level of therapeutic security increased, and increasing percentages of patients with lengths of stay above 1 and 5 years.

Within these designated levels of security, a distinction can be made between the 744 open beds in hospital settings (median cross-sectional length of stay 55.0 95% CI 45.5–64.5) and the 121 RMNH places providing 24 h nursed care in community settings (median cross-sectional length of stay 1,257.0, 95% CI 971.8–1,542), log rank X^2^ = 187.9, df = 1, p < 0.001.

#### Other analyses

‘Forensic’ patients could be found occupying registered mental nursing home beds in the community (13/121, 10.7%) and in low secure units (16/63, 25.4%), while civilly detained patients were found in medium (27/86, 25.4%) and high (7/71, 9.9%) secure beds.

In order to explore any confounding effects, a Cox regression analysis was performed with all cases included. Cross-sectional length of stay was the dependent variable and all six variables were entered as covariates. Using forward stepwise regression (likelihood ratio) the algorithm converged at the third step and generated a model containing only commissioning status (general or forensic), designated length of stay (acute, medium term, long term) and designated level of therapeutic security (open, low, medium, high). The remaining three variables gender, secure or open, and legal status (informal, civil, forensic) were eliminated (Table 8). The omnibus test of model coefficients was X^2^ = 815.1, df = 6, p < 0.001 and change from the previous step was X^2^ = 11.1, df = 1, p < 0.001. Repeating this with legal status divided into four by subdividing forensic into unrestricted and restricted made no difference. Backwards stepwise likelihood ratio yielded the same results.

A linear regression analysis was also performed, using cross-sectional length of stay as the dependent variable and the same six categories gender, general or forensic designation of the bed as commissioned, whether the bed was open or secure, whether the bed was designated acute, medium term/rehabilitation or long term, legal status (informal, civil or forensic), and level of therapeutic security (open, low, medium or high secure) as independent variables. This yielded a model with adjusted r^2^ = 0.275. Gender and legal status did not contribute significantly to the model, while forensic or general status (t = −4.8, p < 0.001), secure or open placement (t = −2.4, p = 0.017), level of therapeutic security (t = 5.35, p < 0.001) and designated length of stay (t = 16.5, p < 0.001) all had significant effects on length of stay. As the normal probability plot of the regression standardised residual and the scatter plot indicated a significant deviation from normality and the scatter plot systematically deviated from central, this analysis has not been relied on.

## Discussion

### Key results

The census data demonstrated that designated uses such as acute, rehabilitation and slow-stream differentiated short term, medium term and long term patient populations at least as measured cross-sectionally on a census date. Designated levels of therapeutic security such as open, low, medium and high secure and commissioning status (general or forensic) also appear to influence length of stay. This was in keeping with the objectives of the study since we had hypothesised that designated lengths of stay would follow the same pattern of distinction between units or services according to length of stay. Gender and legal status seemed to have effects that were confounded by other factors, notably whether the bed was commissioned by forensic or general funding, the level of therapeutic security and most powerfully, the actual designation of the bed as short, medium or long term.

### Limitations

The cross-sectional mean or median LOS does not reflect the speed of discharge of short-stay patients. Nor does cross sectional LOS indicate what proportion of admissions will become long-stay [20]. However we believe that this cross-sectional study design has some limited advantages over prospective studies of completed length of stay or 12 month mean completed (discharged) length of stay. The findings here may be specific to London and the National Health Service at the end of the 1990s, and more particularly to the situation a decade after the closure of the asylums and the shift to a community mental health service.

This census study necessarily used cross-sectional data. The service characteristics and legal variables identified were significantly associated with cross-sectional length of stay but no causal inferences can be made since only a prospective study could establish causation.

The process of admission, treatment and discharge in new community based service models is likely to take many years to reach an equilibrium [21, 22]. Such evaluations are further complicated by successive changes to the service models, legal structures and patient care pathways that have succeeded the historical institutional model.

It is not apparent from this analysis whether there were separate parallel pathways operating for forensic and general patients from hospital to the community. It is also likely that there were other mentally disordered persons in prisons awaiting psychiatric beds at the time of the survey, who are not included in this data.

### Length of stay

Length of stay may be calculated from completed lengths of stay in any year, however this is inherently biased by the omission of long-stay patients from such samples. A prospective study of completed length of stay is the least biased way to study the characteristics of patients that determine length of stay, and is the only valid way to study the influence on length of stay of purely clinical factors such as diagnosis and treatment. However a prospective study would take many years to complete, by which time many legal and policy determinants would have changed. Cross-sectional length of stay tends to under-estimate the numbers of short stay patients passing through hospital beds in any period but will include longer stay patients. Cross-sectional census data yields useful information about operational matters concerning the organisation and management of what has become a virtual institution in the community. A cross-sectional study such as this has the advantage of identifying the factors that are relevant now. For practical purposes cross-sectional studies allow health service managers and commissioners to gauge the current pattern of service provision and aspects of service use relevant to longer term patients.

When frequencies of length of stay are plotted against time the distribution has been shown to be similar to an exponential decay curve [20, 23]. Length of stay does not follow a normal distribution and therefore neither the cross-sectional or completed mean lengths of stay are the best measures of central tendency as the mean is easily influenced by outliers [20]. The median and mode are also measures of central tendency [20]. The mode is the most frequent length of stay (LOS), and has been shown to be 1 day in a number of modern studies of completed length of stay. Such a short completed LOS would imply that the most common type of admission is for brief crisis interventions. This figure does not appear to reflect the needs of an acute psychiatric admission service and may represent the lack of community crisis resolution resources. As a measure the modal completed length of stay does show something about what is happening in a service, but as a measure of central tendency it is of limited value and lacks sensitivity. The median is the middle number in an ordinal sequence. It is not as influenced by outliers. For example in an overview of all admissions to English psychiatric hospitals for a year in 1999, the median completed length of stay was 15 days [24]. This figure has been replicated in other studies in the UK [20, 23] and Ireland [25]. This level of repeatability suggests that it is quite a robust statistic. It is notable therefore that when analysing the cross-sectional length of stay in this survey, the mean cross sectional LOS was 966.2 days with median cross-sectional LOS 119 days and mode 5 days. Overall 43.5% had cross sectional lengths of stay greater than 365 days. Simply considering completed lengths of stay would give a very poor guide to the pattern of use of most psychiatric beds. Table 6 illustrates that although forensic beds accounted for 17% of beds and had a cross-sectional mean LOS 2,272.6 days (95% CI 1,906.6–2,638.6) and cross-sectional median LOS 1,367.0 (95% CI 965.0–1,768.9) the general psychiatric beds when including registered mental nursing home beds in the community also had long cross-sectional mean and median LOS, though not as long as for forensic patients. Similarly secure beds have longer lengths of stay, but open beds also have much longer cross-sectional lengths of stay than might be expected from published figures for completed lengths of stay. While it is true that most psychiatric hospital admissions are now very short [20, 23–25], many patients in hospital and community registered mental nursing home beds at the time of this survey had been in hospital over 1 year and even over 5 years. A popular misconception may have developed about lengths of stay in modern services based on consideration only of completed lengths of stay, and possibly also based on the omission of forensic beds from some published series.

### New long stay patients

Length of stay in psychiatric hospital services is reported to be dependent on a number of factors including patient age, diagnosis, co-morbid substance misuse problems, number of previous admissions [26], duration of illness, physical health [27] and accommodation status. This survey suggests that forensic status and need for therapeutic security are robust determinants of length of stay. ‘New long stay’ patients are defined in the UK national audit as those with admissions lasting between 6 months and 3 years [7]. Table 6 shows that the median cross-sectional length of stay for designated rehabilitation/medium term beds (19% of all beds) is 387 days, indicating that more than half the beds are occupied by patients with a cross-sectional length of stay over a year, while designated long term beds (30% of all beds) had a median length of stay of 1,692 days or 4.6 years.

New long stay patients continue to accrue, despite the movement towards community based care [4]. A study by Cowan and Walker [28] found that a small number of long-stay admissions took up 12% of available admission ward capacity over 3 years, placing demands on the inpatient service greatly disproportionate to their numbers. Similarly it has been shown that many patients in acute admission units are inappropriately placed new long stay patients [25]. A number of studies found that young men with psychosis and elderly women with dementia are the predominant groups in the new long stay category [7, 24]. It may be their cognitive or physical disabilities, challenging behaviour, lack of rehabilitation beds, appropriate housing in the community or the lack of specialist services that prevents their discharge. These same factors may also be relevant to risk of violence and need for therapeutic security. With inception rates for new long stay admissions of 3.3 per 100,000 population in the UK national audit [7] and 2.0 per 100,000 in a modernised district [28], it would appear that a number of new-long stay beds need to be created annually. This survey suggests that at present much of the need for new long stay beds is accounted for by patients accessing beds through forensic routes because of a need for therapeutic security, usually directed by the courts. Predicting future needs must include planning for these new long stay patients in order to ensure that acute admission beds are free to serve the purpose for which they were intended and that the needs of new long-stay patients are appropriately met [4]. Planning should take account of the need for appropriate levels of therapeutic security as well as appropriate lengths of stay.

### Bed usage as a dynamic process

We have not been able to study the pathways by which patients arrived at their placements on the census date, beyond the simple division into civil or forensic legal status and general or forensic commissioning status. Pathways into care and pathways through multiple health, justice and social agencies are shown to be complex because of the wide distribution and mixing of ‘civil’ and ‘forensic’ patients across designated uses and levels of therapeutic security (Tables 2, 3, 4, 6).

In a more theoretical sense, it has been shown that the use of hospital beds, including psychiatric hospital beds, is a dynamic process and can be modelled if it is assumed that an equilibrium exists. The distribution of lengths of stay in cross-sectional data can then be fitted by an equation in which a mixed exponential can be interpreted as a number of ‘phases’ e.g. short term, medium term and long term phases, each characterised by parameters such as the half-life in that phase, the calculated number in that phase and the proportion of all admissions who are either discharged from each phase or transition to the next [21, 22, 29]. This form of analysis will be developed in a subsequent paper. In practice when equilibrium is slow to develop, frequent changes of policy and organisation mean that equilibrium is seldom reached and no evaluation can be completely reliable.

### Interpretation

The findings here could be interpreted as showing that by the late 1990s forensic beds and forensic care pathways had become the main route of access for medium to longer periods of hospital treatment. Forensic commissioned beds accounted for 17% of all beds, secure beds were 19.9% of the total and forensic legal status accounted for 14.5% of beds. More analysis is required to discover whether patients admitted to medium or high or secure settings were likely to be discharged from those settings to a less secure place or directly to a community place. These pathways through care remain largely unquantified and unstudied at the systems level. There is little in the way of an evidence base for how to optimise length of stay in forensic and secure settings. The systematic study of competing models such as parallel or integrated care pathways remains unexplored, although there is evidence that forensic patients subject to conditional discharge are less likely to reoffend than others. We believe this data draws attention to the need for population based prospective observational cohort studies of pathways into psychiatric care to underpin future policy developments in mental health and criminal justice, and commissioning practice in mental health services. These should use standardised assessments of need for therapeutic security [30] and readiness for moves to less secure places [31].

### Generalisability

This study describes the disposition of all psychiatric beds for a defined catchment area of 1.2 million. A ‘whole system’ approach is essential in order to study a population based public service. Had we excluded forensic patients, or had we considered only patients within the catchment area excluding those placed elsewhere, the survey would have under-estimated the number of beds occupied by patients with long cross-sectional lengths of stay. The use of cross-sectional length of stay ensured that longer stay patients were not omitted from calculations of length of stay and calculation of median length of stay provided a reliable estimator for comparative purposes. We were able to capture the numbers and cross-sectional lengths of stay of those in registered mental nursing home beds in the community, equivalent to high support community places. This element of the present study may be the key to generalizability—in other populations, the contribution of such patient groups to length of stay may be missed. Yet we believe these to be the most disabled and the most needy, due to severe, enduring and disabling mental disorders.

## Conclusions

In the years after the closure of asylum style psychiatric hospitals, the means of delivering care to persons with severe, enduring and disabling mental disorders has developed in an organic way that does not reflect any one policy initiative. A variety of short term, acute services have arisen from modern psychiatric practice including acute admission wards and short term psychiatric intensive care units. Specialist services for eating disorders and perinatal care were also established. Longer term care for those with relatively low dependency needs remained a substantial resource. New long stay places appeared to be accessed mainly via forensic routes. These long stay places were not abolished by closing the asylums but instead formed a virtual asylum, with registered mental nursing homes dispersed within the catchment area and forensic high, medium and long term low secure units dispersed regionally and nationally [32]. The criteria for accessing these services [13, 15, 30] or for exiting them [31] have received little scientific interest until recently. The pathways connecting these services were poorly defined and difficult to study. This analysis has not taken account of the numbers of severely mentally ill persons from the same catchment area in courts [33] and prisons [10] either awaiting general psychiatric or forensic hospital beds or simply lost to psychiatric services. Future research might usefully consider whether services are meeting the needs of people with severe, enduring and disabling mental illnesses, or are reactive to changes in legal processes and public policy. It has been shown that changes in prison numbers arise from changes in political policy, not from changes in demography or geographic or economic characteristics of the population [34]. The same may be true of the psychiatric institutional population.
